# Refugee Health: An Ongoing Commitment and Challenge

**DOI:** 10.3390/ijerph15010131

**Published:** 2018-01-13

**Authors:** Jimmy T. Efird, Pollie Bith-Melander

**Affiliations:** 1 Centre for Clinical Epidemiology and Biostatistics (CCEB), School of Medicine and Public Health, The University of Newcastle (UoN), Callaghan, NSW 2308, Australia; 2 Alternatives in Action, Oakland, CA 94610, USA; polliebith@gmail.com

**Keywords:** refugee health, exploitation, lack of resources, struggles of cultural transition, hygiene/sanitation, disease risk, early marriage

## Abstract

Refugees represent a diverse group of displaced individuals with unique health issues and disease risks. The obstacles facing this population have their origins in war, violence, oppression, exploitation, and fear of persecution. Regardless of country of origin, a common bond exists, with refugees often confronting inadequate healthcare resources, xenophobia, discrimination, and a complex web of legal barriers in their new homelands. In many cases, the plight of refugees is multigenerational, manifesting as mental health issues, abuse, poverty, and family disruption. The health trajectory of refugees remains an ongoing commitment and challenge.

## 1. Introduction

While civilization has a long history of major displacement crises, recent times have witnessed alarmingly high levels of families and individuals seeking refuge and protection from hostile world conditions. At latest count, nearly 66 million people have been forcibly displaced from their homes owing to conflict or persecution, with an additional 10 million living in statelessness [1]. This amounts to approximately 20 new refugees every minute, over half of whom are under the age of 18. The healthcare needs of this population are complex. Solutions are frequently mired in political disarray, misguided national security concerns or divergent views regarding the best path forward. The multicultural and multilingual composition of refugees further confounds preparedness efforts and crises management. Increasingly, refugees are encountering extended delays and suboptimal responses in their resettlement to a safe haven, often influenced by populism and partisan ideology rather than evidence-based public health data. 

## 2. Moving Toward a Solution

Solving the healthcare requirements of a vulnerable refugee population merits a flexible framework. This is important to facilitate understanding by health system providers and to elicit an optimal response by receiving government agencies. Originating from various regions of the world, the needs of refugees must be carefully considered in the context of cultural and economic differences with their host countries. Diseased burden and risk is best placed in a proper public health context, supported by legitimate scientific facts and realistic healthcare policy approaches, in order to achieve a lasting solution. 

The consideration of a wide range of topics is needed to better inform decision makers and the medical community [2,3,4,5,6,7,8,9,10,11,12,13,14,15,16,17,18,19,20,21,22,23,24,25]. This includes, but is not limited to: Health screening examinations; community-based/multi-lingual health education and resources for refugees; improved awareness of refugee health disparities; disease tracking and reporting systems; multidisciplinary response to disease outbreaks; refugee utilization or lack thereof of healthcare resources/insurance; barriers to healthcare; health selection/trajectory of refugees; hematologic genetic disorders; chronic and mental health issues; abuse; discrimination; exploitation; infectious diseases; immunization strategies; environmental exposures related to disease; hygiene and sanitation; healthy lifestyle choices; illegal activities/injuries (assault, rape, battery); and assimilation analysis. Healthcare concerns relating to economic challenges, language barriers, the struggles of a cultural transition, geographic origin, and refugee camp living conditions are other topics that may have a significant impact on refugee resettlement and their healthcare needs. 

## 3. Conclusions 

The United States, Europe, China, and Israel are some of the major world powers that have played a leading role in providing humanitarian aid to a world in crisis. Recognizing the healthcare needs of refugees is consistent with the belief that all people deserve equitable access to quality medical treatment and prevention. Facilitating the cooperation between countries, encouraging open political dialog, and developing strategies to increase philanthropic involvement from the private/non-governmental sector are key to achieving long-term solutions for the health and safety of displaced individuals throughout the world.
